# Cafeteria diet increased adiposity in comparison to high fat diet in young male rats

**DOI:** 10.7717/peerj.6656

**Published:** 2019-04-05

**Authors:** Yucel Buyukdere, Atila Gulec, Asli Akyol

**Affiliations:** Department of Nutrition and Dietetics, Hacettepe University, Ankara, Turkey

**Keywords:** Cafeteria diet, High fat diet, Obesity, Adiposity, Rat

## Abstract

**Background:**

Dietary intervention studies in animal models of obesity are crucial to elucidate the mechanistic effects of specific nutrients and diets. Although several models of diet induced obesity have been examined in rodents to assess obesity, there are few studies that have researched influence of different high fat and/or westernized diets. The aim of this study was to compare a high fat diet and a cafeteria diet on obesity related biochemical and physiological parameters in young male rats.

**Methods:**

Five week old Wistar male rats were fed a control chow diet (C), butter-based high fat diet (HF) or cafeteria diet (CAF) for twelve weeks. In HF, 40% of energy came from fat and this ratio was 46% in CAF. CAF composed of highly energetic and palatable human foods along with chow diet. At the end of the feeding protocol all animals were culled using CO_2_ asphyxia and cervical dislocation after an overnight fasting.

**Results:**

Total energy and fat intake of CAF was significantly higher than C and HF. CAF was more effective in inducing obesity, as demonstrated by increased weight gain, Lee index, fat depot weights and total body fat in comparison to C and HF. Despite increased adiposity in CAF, plasma glucose, insulin and HOMA-IR levels were similar between the groups. Plasma leptin and cholesterol levels were markedly higher in CAF than C and HF.

**Discussion:**

We have demonstrated that there are differential effects of high fat diet and cafeteria diet upon obesity and obesity-related parameters, with CAF leading to a more pronounced adiposity in comparison to high fat diet in young male rats. Future studies should consider the varied outcomes of different diet induced obesity models and development of a standardized approach in similar research practices.

## Introduction

Obesity is a worldwide epidemic increasing at an alarming rate. Obesity is multifactorial in nature but is primarily due to positive energy balance and unhealthy dietary habits (Lifshitz & Lifshitz, 2014). An elevated adiposity develops when energy consumption is in excess of energy expenditure. The resulting increase in body mass index raises the risk for cardiovascular disease, diabetes, hypertension, dyslipidemia and specific types of cancers (Neeland, Poirier & Després, 2018). Moreover, metabolic disease physiological parameters that were once assumed to be limited during the later stages of life are now developing in children with overweight and obesity (Liang et al., 2015). Clearly, there is an immediate requirement to elucidate the mechanisms by which obesity develops and to present novel interventions for prevention of this disorder (Zolotarjova, Ten Velde & Vreugdenhil, 2018).

Dietary studies in animal models are crucial to examine the mechanisms involved in the progression of obesity (Bagnol et al., 2012). In this sense, diet induced obesity models are one of the most frequently used model for studying obesity (Giles, Jackman & MacLean, 2016). Diet-induced obesity models, including high-fat diets and cafeteria diets, have been employed to study obesity in rats (Crew, Waddell & Mark, 2016; Nguyen et al., 2017; Xia et al., 2018). These models have exhibited evidence of the role of overnutrition in energy homeostasis, body weight regulation and adiposity but there is a paucity of data regarding their nutritional composition (Hariri & Thibault, 2010). For instance, high fat diets can be comprised of various or purified fat sources in distinct proportions (Buettner et al., 2006; Dziedzic et al., 2007; Crescenzo et al., 2015). Therefore, the resulting phenotype may represent different physiological and biochemical parameters poses a challenge to drawing comparisons between the studies (Tracy et al., 2015).

The cafeteria diet consists of highly energetic and highly palatable human foods along with chow diet to trigger diet induced obesity in laboratory animals (Sclafani & Springer, 1976). Cafeteria diet is relatively a robust alternative to the feeding of purified high-fat diets to induce obesity as it prevents the use of very high intakes of a particular type of fat while inducing continuous hyperphagia and increased energy intake (Lalanza et al., 2014; Gomez-Smith et al., 2016). These diets also provide a substantial amount of sugar and salt, which increases the appetite (Oliva et al., 2017). Despite its useful properties, it has been reported that cafeteria diet may produce some variation in foods and nutrients consumed between animals in the same group due to different food choice of animals (Moore, 1987). Conversely, cafeteria diet has also non-standardized practices as researchers use different “junk foods” when forming the cafeteria diet (Shafat, Murray & Rumsey, 2009; Akyol, Langley-Evans & McMullen, 2009). On the other hand, it has been indicated that these outcomes are more closely aligned with dietary patterns observed in humans than conventional purified high-fat diets (Martire et al., 2014). The use of Western dietary patterns as a measure of exposure in human studies of diet and metabolic syndrome relationships is shown to provide a stronger basis of study (Zeeni et al., 2015).

To date, several studies have shown the detrimental effects of high fat diets or cafeteria diets in rats but direct comparisons between these models have not been examined (Sampey et al., 2011). Recently, Oliva et al. (2017), reported that cafeteria diet exerted a greater impact upon food intake due to its sugar and salt content than a classical model of high fat diet based on coconut oil. Previous studies also reported similar results with lard based high fat diets (Sampey et al., 2011; Bortolin et al., 2018). To date, a butter based high fat diet has not been compared to cafeteria diet in rat studies. In addition, most of the studies that examined the difference between high fat and cafeteria diets used adult rats. Therefore, the aim of this study was to compare the metabolic responses induced by a butter-based high fat diet and cafeteria diet in younger rats.

## Materials & Methods

### Animals and diets

The experiments were performed under the license from the Ethics Committee of Hacettepe University, Ankara, Turkey, number: 2016/51. All animals were housed individually in plastic cages and subjected to a 12 h light-dark cycle (08.00 and 20.00) at a temperature of 20–22 °C and 45% humidity. The animals were housed on shavings and had *ad libitum* access to food and water at all times. After one week of habituation period, male Wistar rats (aged 5 weeks) were randomly allocated to be fed either a control chow diet (C; *n* = 6), high fat diet (HF; *n* = 6) or cafeteria diet (CAF; *n* = 6) for twelve weeks. Experimenters and data analyst were not blind to treatment. The C and HF diets met the protein (20% casein) and micronutrient content of the AIN-93G diet (ARDEN Research & Experiment, Ankara) (Reeves, Nielsen & Fahey Jr, 1993). The carbohydrate and fat content of total energy were as follows: 4.1% fat and 75.9% carbohydrate in C and 40% fat and 40% carbohydrate in HF. The fat content of HF composed of butter (89% percentage of total fat) and soy oil (11% of total fat) and these were added to the diet during clusterization of the components. CAF included peanuts, cheese, potato chips, variety of biscuits, corn chips, crackers and variety of chocolates along with control chow diet. Five of the cafeteria foods were provided in a bowl on the cage floor daily in excess quantities. The foods provided were altered daily, to maintain variety, by replacing three of the foods with new items. Hence the animals did not receive the same foods for more than two consecutive days at a time. The chow diet and cafeteria diet foods were individually weighed in and out of the cage between 09.00 and 10.00 h daily. Daily intakes of energy, macronutrients and micronutrients were calculated from the manufacturers’ data for cafeteria diet including peanuts. The resulting cafeteria diet provided an average of 41.7% energy from carbohydrates, 11.4% from protein and 46.9% from fat. CAF and HF diets were not isocaloric. At the end of the feeding protocol all animals were culled using CO_2_ asphyxia and cervical dislocation after overnight fasting. Blood samples were taken by cardiac puncture, and major organs were weighed and sampled.

### Body composition

Body composition was determined in all animals by chemical analysis. Whole carcasses were oven-dried to determine the body water content as previously described (Langley & York, 1990). The dried carcasses were homogenized and sampled for estimation of N content by the Kjeldahl method and for fat content by Soxhlet extraction.

### Plasma and liver metabolites

Plasma glucose (Cayman Chemical Company, USA), insulin (Elabscience Biotechnology, China), leptin (BioVendor Laboratory Medicine, Czech), total cholesterol (Shanghai Sunred Biological Technology, China) and triglycerides (Shanghai Sunred Biological Technology, China) analyses were performed using a commercially available ELISA kits. Livers triglyceride content was analysed using a commercially available ELISA kit (Shanghai Sunred Biological Technology, China) according to manufacturer’s instructions.

### Statistical analyses

All data were analysed using the Statistical Package for Social Sciences (version 16; SPSS, Inc., Chicago, IL, USA). The effect of diet on metabolic outcomes was assessed using a general linear model ANOVA. Where longitudinal data were available (for example, weekly body weights or energy intake), the week of study (treatment time) was used in a repeated-measures analysis. Values are expressed as mean values with their standard errors. *p* < 0.05 was considered statistically significant. Post hoc testing (Tukey’s test) was applied for the main effects of the diet. Power analysis indicated that 6 animals per group was sufficient to detect a minimum 12% energy intake difference with a power of 80% and alpha 0.05 (Akyol, Langley-Evans & McMullen, 2009).

## Results

**Figure 1:**
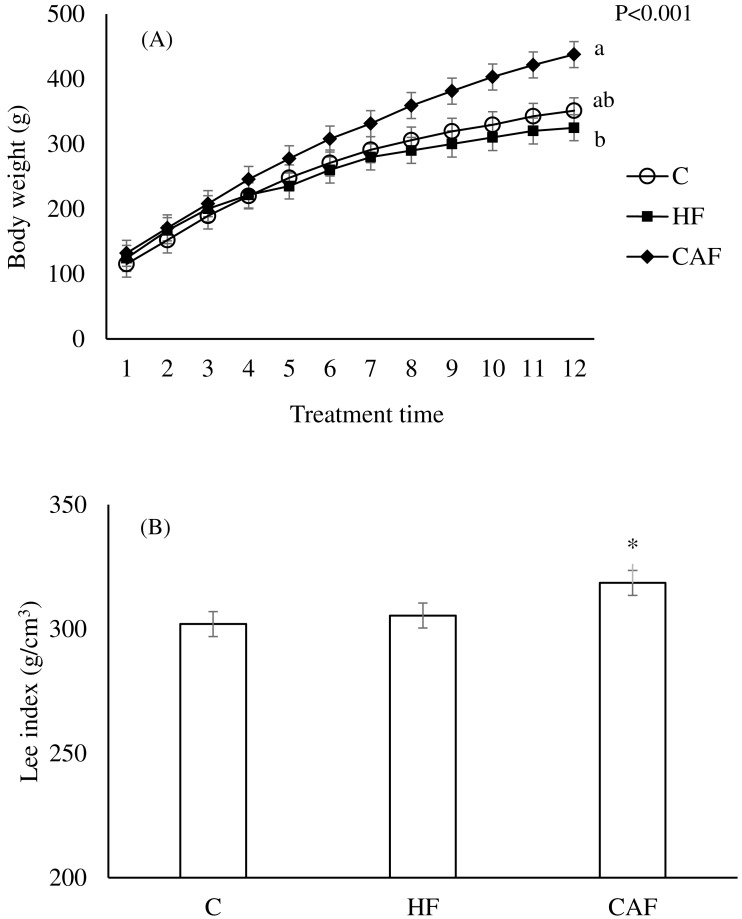
Body weight changes during the study weeks and Lee index at the end of the study in rats. Body weight changes during the study weeks in rats fed a control (C, *n* = 6), high fat (HF, *n* = 6) or cafeteria diet (CAF, *n* = 6). Repeated measures ANOVA indicated that diet body weight changes was significantly different between groups over weeks (Diet and treatment time interaction, *P* < 0.001). Post hoc test showed that weight gain during the study weeks was significantly higher in animals fed CAF in comparison to HF. Different letters indicate significant differences between groups. (B) Lee index at the end of the study in rats. ANOVA indicated that diet (*P* = 0.021) significantly influenced Lee index. * Post hoc test showed that Lee index was significantly higher in animals fed CAF in comparison to HF. Values are means, with standard errors represented by vertical bars.

All rats gained weight over time (Fig. 1A). Overall, diet (*p* = 0.004) significantly influenced the body weights of the animals. Body weight changes was significantly different between groups over weeks (Diet and treatment time interaction, *P* < 0.001). Rats fed the CAF during the treatment time had a significantly increased weight gain in comparison with those fed the HF (C, 261.28 ± 3.88 g; HF, 235.41 ± 3.89 g; CAF, 306.28 ± 3.88 g). There were no differences between C and HF groups and C and CAF groups. At the end of the study, rats fed the CAF had a significantly higher Lee index when compared to C and HF (C, 302.02 ± 3.05 g/cm^3^; HF, 305.45 ± 3.06 g/cm^3^; CAF, 318.56 ± 3.068 g/cm^3^; *p* = 0.021). The Lee index of C and HF were similar (Fig. 1B).

**Figure 2:**
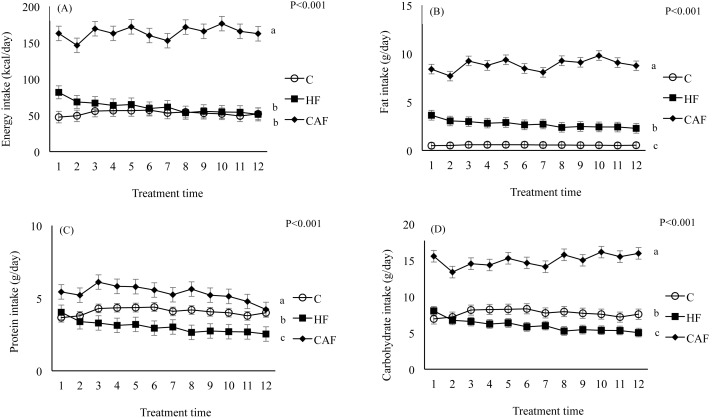
Energy and nutrient intakes during the study weeks in rats fed a control (C, *n* = 6), high fat (HF, *n* = 6) or cafeteria diet (CAF, *n* = 6). Different letters indicate significant differences between groups. (A) Energy intake. Repeated measures ANOVA indicated that diet (*P* < 0.001) significantly influenced energy intake of the animals. Energy intake was significantly different between groups over weeks (Diet and treatment time interaction, *P* = 0.034). Post hoc test showed that energy intake during the treatment time was significantly higher in animals fed CAF in comparison to C and HF. (B) Fat intake. Repeated measures ANOVA indicated that diet (*P* < 0.001) significantly influenced fat intake of the animals. Fat intake was significantly different between groups over weeks (Diet and treatment time interaction, *P* = 0.047). Post hoc test showed that fat intake during the treatment times was significantly higher in animals fed CAF in comparison to C and HF and significantly higher in animals fed HF in comparison to C. (C) Protein intake. Repeated measures ANOVA indicated that diet (*P* < 0.001) significantly influenced protein energy intake of the animals. Protein intake was significantly different between diet groups over weeks (Diet and treatment time interaction, *P* < 0.001). Post hoc test showed that protein intake during the treatment time was significantly higher in animals fed CAF in comparison to C and HF and significantly lower in animals fed HF in comparison to C. (D) Carbohydrate intake. Repeated measures ANOVA indicated that diet (*P* < 0.001) significantly influenced carbohydrate intake of the animals. Carbohydrate intake was significantly different between diet groups over weeks (Diet and treatment time interaction, *P* = 0.011). Post hoc test showed that carbohydrate intake during the treatment time was significantly higher in animals fed CAF in comparison to C and HF and significantly lower in animals fed HF in comparison to C).

Figure 2 shows the energy and nutrient intakes of rats during the study protocol. The effects of cafeteria feeding were also reflected in the data showing energy intakes and macronutrients. Energy intake was significantly different between groups over weeks (Diet and treatment time interaction, *P* = 0.034). Rats fed the CAF had a significantly increased energy intake in comparison with those fed the C and HF (C, 53.00 ± 1.66 kcal/day; HF, 61.26 ± 1.66 kcal/day; CAF, 163.83 ± 1.66 kcal/day). Furthermore, energy intake of HF was similar to C. The impact of diet on fat intake of the animals was apparent as rats fed the CAF and HF had a significantly increased fat intake in comparison with those fed the C (C, 0.55 ± 0.10 g/day; HF, 2.72 ± 0.10 g/day; CAF, 8.83 ± 0.10 g/day). (Diet and treatment time interaction, *P* = 0.047). Similar to energy intake, fat intake of CAF was significantly higher than HF. Protein intake was significantly different between diet groups over weeks (Diet and treatment time interaction, *P* < 0.001). CAF had significantly higher protein intake than C whereas HF had significantly lower protein intake than C (C, 4.08 ± 0.06 g/day; HF, 3.02 ± 0.06 g/day; CAF, 5.34 ± 0.06 g/day). Carbohydrate intake was also significantly different between diet groups over weeks (Diet and treatment time interaction, *P* = 0.011). This resulted in lower intake of carbohydrates in HF and higher intake in CAF when compared to C (C, 7.73 ± 0.16 g/day; HF, 6.02 ± 0.16 g/day; CAF, 15.02 ± 0.16 g/day).

**Figure 3:**
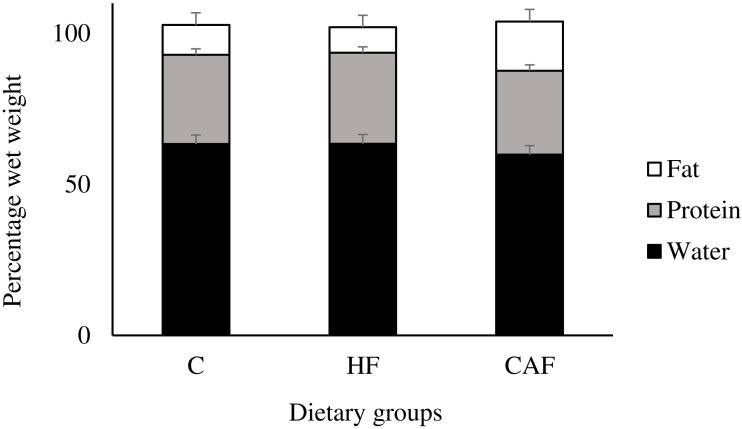
Body fat, protein and body water in rats fed a control (C, *n* = 6), high fat (HF, *n* = 6) or cafeteria diet (CAF, *n* = 6). ANOVA indicated that diet significantly influenced body fat (*P* < 0.001) and body water (*P* < 0.001) of the animals. Post hoc test showed that body fat was significantly higher in animals fed CAF in comparison to HF and C whereas body water was significantly lower in animals fed CAF in comparison to HF and C. Body protein levels were similar among the groups. Values are means, with standard errors represented by vertical bars.

Major organ weights and fat depots relative to body weight of the animals are shown in Table 1. Diet influenced all of the major organ weights and fat depots significantly (*p* < 0.05). According to this, liver weight was significantly lower in HF and CAF than C (*p* = 0.044). Diet exerted a distinct influence on brain weight as HF had significantly increased brain weight than CAF (*p* = 0.022). Data of the average weights of right and left kidneys relative to body weight showed that CAF had significantly reduced kidney weight in comparison to HF (*p* = 0.005). Kidney weights were similar between C and HF. Cafeteria feeding for twelve weeks resulted in a pronounced adiposity in rats since peri-renal (*p* < 0.001) and epididimal (*p* < 0.001) fat depots were found to be significantly increased in CAF when compared to HF and C. These fat depots were similar between C and HF. In addition, subcutaneous brown adipose tissue (*p* = 0.016) was significantly heavier in CAF than C, alone. These modifications in adipose tissue were also observed in body composition of the animals (Fig. 3). There was a significant effect of diet on body fat (*p* < 0.001) and body water (*p* < 0.001) of the animals which indicated an increase in body fat (C, 9.93 ± 0.82%; HF, 8.43 ± 0.82%; CAF, 16.37 ± 0.82) and a reduction in body water (C, 63.33 ± 0.53%; HF, 63.47 ± 0.52%; CAF, 59.88 ± 0.53%) of CAF when compared to C and HF. These parameters were similar between C and HF. Body protein percentage did not differ between the study groups (C, 29.53 ± 1.19%; HF, 30.08 ± 1.18%; CAF, 27.68 ± 1.19%).

**Table 1:** Organ size and fat depot mass relative to body weight.

	**Groups**		
Organ and fat depot (% of body weight)	C	HF	CAF
Liver	2.69 ± 0.07^a^	2.45 ± 0.07^b^	2.44 ± 0.07^b^
Brain	0.47 ± 0.03^a^^,^^b^	0.52 ± 0.03^a^	0.41 ± 0.03^b^
Kidney	0.33 ± 0.01^a^	0.33 ± 0.01^a^	0.28 ± 0.01^b^
Peri-renal fat	1.01 ± 0.21^a^	0.74 ± 0.21^a^	3.47 ± 0.21^b^
Epididimal fat	1.31 ± 0.09^a^	1.33 ± 0.09^a^	2.51 ± 0.09^b^
Brown adipose tissue	0.23 ± 0.04^a^	0.35 ± 0.04^a^^,^^b^	0.44 ± 0.04^b^

**Notes.**

Organ size and fat depot mass relative to body weight and absolute values in rats fed a control (C, *n* = 6), high fat (HF, *n* = 6) or cafeteria diet (CAF, *n* = 6).

Mean values with their standard errors.

a,b Mean values with unlike superscript letters were significantly different (*P* < 0.05).

Although they were markedly influenced by the dietary treatment, the HF and CAF-fed rats showed little evidence of metabolic disturbance in glucose homeostasis (Table 2). Plasma glucose, insulin and HOMA-IR levels were unaffected by diet. On the other hand, there was a significant effect of diet (*p* < 0.001) on leptin concentrations which showed that plasma leptin was markedly higher in CAF than C and HF (Table 2). In addition, plasma cholesterol concentration was significantly increased in CAF in comparison to C and HF (*p* = 0.014). The leptin and cholesterol levels were similar between C and HF (Table 2). Although it was not significant, diet exerted a tendency towards significance on triglyceride levels (*p* = 0.074) which indicated that the triglyceride concentration was higher in CAF and HF than C. Moreover, triglyceride level in liver was significantly influenced by diet (*p* = 0.038) and this exhibited an increase in HF and CAF in comparison to C. The triglyceride level in liver was found to be similar between HF and CAF.

**Table 2:** Concentrations of fasting biochemical parameters.

	**Groups**		
Biochemical parameter	C	HF	CAF
Glucose (mg/dL)	114.49 ± 12.26	142.38 ± 12.26	129.14 ± 12.26
Insulin (µIU/mL)	68.08 ± 18.01	52.72 ± 18.01	73.99 ± 17.73
HOMA-IR	17.64 ± 5.70	18.44 ± 5.70	29.03 ± 6.98
Leptin (ng/ml)	1.93 ± 0.70^a^	1.57 ± 0.70^a^	7.24 ± 0.70^b^
Cholesterol (mg/dL)	148.65 ± 7.66^a^	172.93 ± 7.66^a^	184.57 ± 7.66^b^
Triglyceride (mg/dL)	195.50 ± 13.26	234.58 ± 13.26	237.41 ± 13.26
Liver triglyceride (mg/g)	7.78 ± 0.26^a^	8.63 ± 0.26^b^	8.73 ± 0.26^b^

**Notes.**

Concentrations of fasting biochemical parameters in plasma and liver in rats fed a control (C, *n* = 6), high fat (HF, *n* = 6) or cafeteria diet (CAF, *n* = 6).

Mean values with their standard errors.

a,b Mean values with unlike superscript letters were significantly different (*P* < 0.05).

## Discussion

Rodent models of diet-induced obesity are commonly used methods to assess the underlying mechanisms of metabolic disturbances that are present during overnutrition. In contrast to the more preferred purified high fat diets in such studies, the comparable effects of cafeteria diet feeding on young rats remain largely unknown. Few studies support the hypothesis that there may be evident implications for cafeteria diet fed animals in comparison to high fat diet fed animals, in terms of inducing obesity related parameters (Sampey et al., 2011; Higa et al., 2014; Oliva et al., 2017). Therefore, the primary aim of the present research was to compare a high fat diet based on butter and cafeteria diet within a close range of energy percentage coming from fat (40% of energy in HF and 46.9% of energy in CAF) on growth, food intake and obesity parameters in male rats. This is the first study comparing a butter based high fat diet to cafeteria diet in rats during post-weaning period although both diet models have been used in the literature, previously.

We have shown that cafeteria diet feeding induced obesity at a significant increased rate when compared to a high fat diet and triggered persistent hyperphagia and increased energy intakes as a result of the diversity and novelty of the foods presented. Indeed, throughout the study period CAF group showed a continuous higher energy and food intake. These results are in agreement with other studies showing that energy and food intake increase after access to a cafeteria diet in comparison to high fat diets (Sampey et al., 2011; Zeeni et al., 2015; Oliva et al., 2017; Bortolin et al., 2018). The increased energy intakes of the cafeteria diet-fed rats were linked with a significant alteration in the composition of their diets and thus the increased intake of fat, carbohydrate and protein. Although the resulting cafeteria diet provided a substantially higher fat intake with lower carbohydrate and protein intakes compared to other two diets of the study in terms of percentage of energy, this did not lead to consuming lower protein and lower carbohydrate than HF and C groups. As the quantity of food intake was increased in CAF, the animals in this group consumed significantly higher amounts of macronutrients. In addition, HF rats decreased the quantity of the food they consumed, possibly due to a regulatory adaptation to higher energy intake, a situation which was also observed in previous studies (Sampey et al., 2011; Zeeni et al., 2015). These results suggest that high fat diets alone could be a relatively limited option to exceed the threshold that is necessary to alter the normal regulatory mechanisms of food intake. This also implies that the other features of cafeteria diet such as higher sodium, saturated fatty acids, trans fatty acids, sugar and lower fibre and micro nutrients along with high fat contribute to the dysregulation of energy homeostasis.

CAF-fed animals had significantly increased body weights than HF in the current study. Several other studies reported similar outcomes (Sampey et al., 2011; Zeeni et al., 2015; Oliva et al., 2017; Bortolin et al., 2018) except one study which found similar body weights at the end of twelve weeks between control, high fat and cafeteria diets (Higa et al., 2014). Sampey et al. (2011) suggested that the similarity between C and HF could be due to the duration of the study protocol or using relatively aged animals to induce metabolic syndrome. Our data indicated that using younger animals did not change this outcome since starting the feeding protocol after weaning still led to comparable body weights between C and HF. However, an extended duration of the study protocol could have changed the body weight of HF. Nevertheless, none of the fat depots and body fat data exhibited a significant difference between C and HF whereas these parameters were consistent in CAF. These results agree with the finding that HF group predominantly utilised lipids for oxidation and basically turned the disadvantageous low carbohydrate status into a compensative metabolic state (Oliva et al., 2017).

One of the main findings of the current study was that the cafeteria diet feeding induced a metabolic phenotype and obesity-related markers when compared to HF but glucose homeostasis was maintained in all of the study groups. Unlike this outcome, other studies comparing high fat and cafeteria diets reported a pre-diabetic condition (Sampey et al., 2011); hyperglycemia, glucose intolerance and hyperinsulinemia (Higa et al., 2014); greater impairment in glucose homeostasis (Bortolin et al., 2018) and lower glucose tolerance response to a glucose challenge (Zeeni et al., 2015). One study also did not find an apparent difference in glucose levels of control, high fat or cafeteria diet fed rats (Oliva et al., 2017). However, a large body of evidence showed that excessive accumulation of adipose tissue was robustly correlated with the development of glucose intolerance and insulin resistance (Kohlgruber & Lynch, 2015; Rebuffat et al., 2018). Therefore, the lack of an impaired glucose homeostasis in the current study may be due to study period, differences in diet models and rat strain. It was shown that some rodents fed high fat diets tend to exhibit a resistance or suppression in metabolic parameters of obesity in comparison to obesity-prone rodents (Akieda-Asai et al., 2013).

Increased leptin secretion or attenuated leptin sensitivity was reported to be linked with excess triglyceride accumulation in insulin sensitive tissues including liver (Yadav et al., 2013). In the current study leptin levels was dramatically increased in CAF group which indicated a condition of leptin resistance in comparison to C and HF. Although both HF and CAF had significantly higher triglyceride content in liver, leptin resistance status was more distinct in CAF due to increased adiposity and cholesterol levels accompanied with a greater hyperphagia. The lipidemia worsening effect of CAF diet was also shown in a different study in which cholesterol levels increased at a greater extent than HF diet (Zeeni et al., 2015).

The relationship of butter, which is the highest in dairy fat, with chronic diseases was shown in epidemiological studies (Engel & Tholstrup, 2015). The higher saturated fatty acids content of butter is also used in animal models of diet induced obesity in different studies (Hariri & Thibault, 2010; Ressler et al., 2015; Song et al., 2018). However, it is known that the saturated fatty acid composition of butter is lower than other saturated fatty acid dense lipid sources such as coconut oil (Khaw et al., 2018). The moderate effects of HF diet in the current study could be due the fatty acid composition of butter. The effects of different dietary fat sources on food intake, metabolic markers and adiposity levels may vary not just according to the general classification of their main component fatty acids as saturated or unsaturated but probably due to distinct compositions in individual fatty acids and also the foods in which they exist or dietary patterns. These outcomes highlight the requirement for future examination of standardised diet induced obesity models in laboratory animals.

Palatability of the diet is an important determinant of food intake as several studies reported that it promotes overeating which in turn contribute to the development of obesity (Leigh, Lee & Morris, 2018). Despite a wide range of use in rodent models of obesity, purified high fat diets do not reflect a true Westernized diet and therefore may exert an insufficient response in obesity development occasionally (Oliva et al., 2017). For this reason, cafeteria diets are considered to be a more efficient diet induced obesity model, comparatively as they introduce a variety of highly palatable energy dense foods that are widely consumed in Western societies (Ferreira et al., 2018). As reported before in previous studies, current study also exhibited that not all high fat diets induced obesity in young rats while cafeteria diet provided a highly relevant diet induced obesity model in terms of reflecting a human diet. However, cafeteria diet has been criticized for being a non-standardized method due to using varied snacks with different compositions, causing animals to consume different types of foods hence generating different dietary compositions (Bortolin et al., 2018). More specifically, it was suggested that accurate measurements of energy intake were difficult to achieve as it was possible for each animal to prefer an individual selection of foods (Reuter, 2007). Despite these disadvantageous factors, it was also concluded that these criticisms can be overcome by careful use of the feeding protocols and well-controlled experiments (Rothwell & Stock, 1988). Male rats were negatively affected by both HF and CAF although CAF exhibited a more disadvantageous phenotype. The effects of dietary modifications can also be manipulated by rodent type, sex and strain (Buettner, Schölmerich & Bollheimer, 2007). As female rats exhibited a predisposition to increased adiposity, current study aimed to examine male rats to address whether cafeteria diet would still exert the obesity triggering effect (Giles, Jackman & MacLean, 2016).

## Conclusions

In conclusion, the current study showed a more pronounced obesity in response to CAF feeding when compared to high fat diet in young male rats. There is still however, a further requirement for the assessment of the optimised diet induced obesity model in rodents in future studies. In agreement with previous studies, we show that cafeteria diet is a robust method to assess the influence of processed and energy dense foods that are widely consumed in Western diets.

##  Supplemental Information

10.7717/peerj.6656/supp-1 Supplemental Information 1 Raw dataset

## Additional Information and Declarations

### Data Availability

The following information was supplied regarding data availability: The raw measurements are available in the Supplemental File.
